# Sulfate erosion investigation on FRP-confined concrete in cold region

**DOI:** 10.1038/s41598-022-15075-z

**Published:** 2022-06-27

**Authors:** Yongcheng Ji, Yunfei Zou, Wei Li

**Affiliations:** School of Civil Engineering, Northeast Forestry University, Harbin, 150040 China

**Keywords:** Engineering, Civil engineering

## Abstract

Fiber-reinforced polymer (FRP) confined concrete is regarded as an innovative and economical approach for structural repairation. Two typical materials [carbon fiber reinforced polymer (CFRP) and glass fiber reinforced polymer (GFRP)] are selected in this study to investigate the concrete strengthen effect in a severe environment. The resist ability of FRP-confined concrete is discussed when subjected to coupled erosion between sulfate erosion and freeze–thaw cycles. Electron microscopy examines concrete's surface and interior degradation during coupled erosion. The corrosion degree and principle of sodium sulfate are analyzed using pH, SEM electron microscope, and EDS energy spectrum. The axial compressive strength test is used to evaluate the reinforcement of the FRP-confined concrete column, and the stress–strain relationship for various FRP-confined techniques in a coupled erosion environment is obtained. The error analysis is performed to calibrate the experimental test result using four existed prediction models. All observations indicate that the deterioration process of FRP-confined concrete is complicated and dynamic under coupled effect. Sodium sulfate initially increases the initial strength of concrete. However, subsequent freeze–thaw cycles may aggravate concrete fractures, while sodium sulfate further degrades the strength of concrete through the cracking development. A precise numerical model is presented to simulate the stress–strain relationship, which is critical for the design and life cycle assessment of FRP-confined concrete.

## Introduction

As an innovative concrete strengthened method explored since the 1970s, FRP has the advantages of low weight, high strength, corrosion resistance, fatigue resistance, and ease of building^1–3^. It is becoming more common in engineering applications as costs fall, such as Glass fiber (GFRP), carbon fiber (CFRP), basalt fiber (BFRP), and aramid fiber (AFRP), which are the most often used FRPs for structural strengthening^4,5^. The proposed FRP-confined technique may increase concrete performance and avoid premature collapse. However, various external environments in engineering often affect the durability of FRP-confined concrete, resulting in its strength failure.

Some researchers have investigated the concrete stress–strain variation laws with various cross-sectional shapes and sizes. Yan et al.^6^ found that the ultimate stress and strain positively related to the fiber fabric thickness grows. Wu et al.^7^ obtained stress–strain curves for FRP-confined concrete using various fiber types to predict ultimate strain and load. Lin et al.^8^ discovered that the FRP stress–strain models for circular, square, rectangular, and elliptical bars are also highly dissimilar and developed a novel, design-oriented stress–strain model, using width ratio and corner radius as parameters. Lam et al.^9^observed that the uneven lap joint of FRP and curvature contribute to FRP's fracture strain and stress being smaller than the plate tensile test. Furthermore, scholars have studied partial confinement and novel confined techniques based on the different requirements in practical projects. Wang et al.^10^conducted axial compression tests concerning three confined modes, including totally, partially, and unconfined concrete. A stress–strain model is developed and provides confinement effect coefficients for partially confined concrete. Wu et al.^11^ developed a method for predicting the stress–strain relationship of FRP-confined concrete that accounts for size impact. Moran et al.^12^ evaluated confined concrete's axial monotonic compressive performance with FRP helical strip and obtained its stress–strain curve. However, the above researches mainly study the difference between partial and entirely confined concrete. The action of the various parts of the FRP partially confined concrete has not been studied in detail.

In addition, studies have also evaluated the effectiveness of FRP-confined concrete under different conditions in terms of compressive strength, strain change, initial elastic modulus, and strain hardening modulus. Tijani et al.^13,14^ found that the repairability of FRP-confined concrete decreased with the increase of the damage degree through FRP repairing experiments on initial damaged concrete. Ma et al.^15^ studied the effect of FRP-confined concrete columns' initial damage and believed that the damage degree had a negligible effect on the ultimate strength and a significant effect on the lateral and longitudinal strain. However, Cao et al.^16^observed the stress–strain and envelope stress–strain curves of FRP-confined concrete under the influence of initial damage. In addition to investigating the concrete initial damage condition, some studies have also appeared on the durability of FRP-confined concrete under harsh environmental conditions. These scholars studied the degradation of FRP-confined concrete in harsh environments and used damage assessment methods to establish degradation models to predict service life. Xie et al.^17^placed FRP-confined concrete in a hydrothermal environment and found that hydrothermal conditions significantly affected the mechanical properties of FRP, resulting in a gradual decrease in its compressive strength. In the acid-base environment, the interface between CFRP and concrete will be affected by degradation. The fracture energy release rate of the CFRP layer decreased significantly with the increase in immersion time, which eventually led to the failure of the interface specimen^18–20^. In addition, some scholars have also studied the effect of freezing and thawing on FRP-confined concrete. Liu et al.^21^pointed out that CFRP reinforcement has good durability under freeze–thaw cycles based on relative dynamic modulus, compressive strength, and stress–strain relationship. Moreover, a model related to the degradation of concrete mechanical properties is proposed. However, Peng et al.^22^ calculated the service life of CFRP-concrete binders using temperature and freeze–thaw cycle data. Guan et al.^23^ conducted a rapid freeze–thaw test on concrete and proposed an evaluation method for freezing resistance based on the thickness of the damaged layer under freeze–thaw action. Yazdani et al.^24^ investigated the effect of FRP layers on the penetration of chloride ions into concrete. The results show that the FRP layer is resistant to chemical corrosion and can separate the internal concrete from the external chloride ions. Liu et al.^25^ simulated the pull-out test environment of sulfate eroded FRP concrete, established a bond-slip model and predicted the degradation of the FRP-concrete interface. Wang et al.^26^ established a stress–strain model of FRP-confined sulfate-eroded concrete through uniaxial compression tests. Zhou et al.^27^ studied the damage of unconfined concrete caused by the coupling of salt-freeze–thaw cycles and used the logistic function to describe its degradation mechanism for the first time. These studies have made considerable progress in evaluating the durability of FRP-confined concrete. However, most researchers have focused on simulating the environment with erosion under a single adverse condition. Concrete is commonly damaged due to coupling erosion caused by various environmental conditions. These coupled environmental conditions have a severe deterioration effect on the performance of FRP-confined concrete.

Sulfates and freeze–thaw cycles are two typical significant parameters to impact the durability of concrete. FRP-confined technology has the potential to enhance the performance of concrete. It is widely used in engineering and research but has its limitations at the current time. Few types of research focus on the endurance of FRP-confined concrete concerning sulfate corrosion in cold regions. The erosion process of fully-confined, semi-confined, and unconfined concrete under the coupled action of sodium sulfate and freeze–thaw deserves more exploration, especially the new semi-confined technique described in this paper. The strengthening effects on concrete columns are also investigated by exchanging the sequence of FRP confinement and erosion. The electron microscope, pH test, SEM electron microscope, EDS energy spectrum analysis, and uniaxial mechanical test are all conducted to demonstrate the micro and macro changes of specimens caused by linked erosion. Additionally, this study discusses the law governing the stress–strain relationship generated by uniaxial mechanical testing. The experimental test's ultimate stress and strain value are verified with error analysis, which uses four existing ultimate stress–strain models. The presented model can adequately predict the ultimate strain and strength of the material, which is helpful for future FRP reinforcement engineering practice. Finally, it also serves as the conceptual foundation for the idea of salt frost resistance in FRP concrete.

## Experimental program

This study evaluates the deterioration of FRP-confined concrete using the sulfate solution corrosion couped with freeze–thaw cycles. The micro and macro changes caused by concrete erosion are demonstrated using a scanning electron microscope, a pH test, an EDS energy spectrum analysis, and a uniaxial mechanical test. In addition, the mechanical properties and stress–strain variations of FRP-confined concrete subjected to coupled erosion are investigated using axial compression experiments.

### Material and concrete mix design

**Figure 1:**
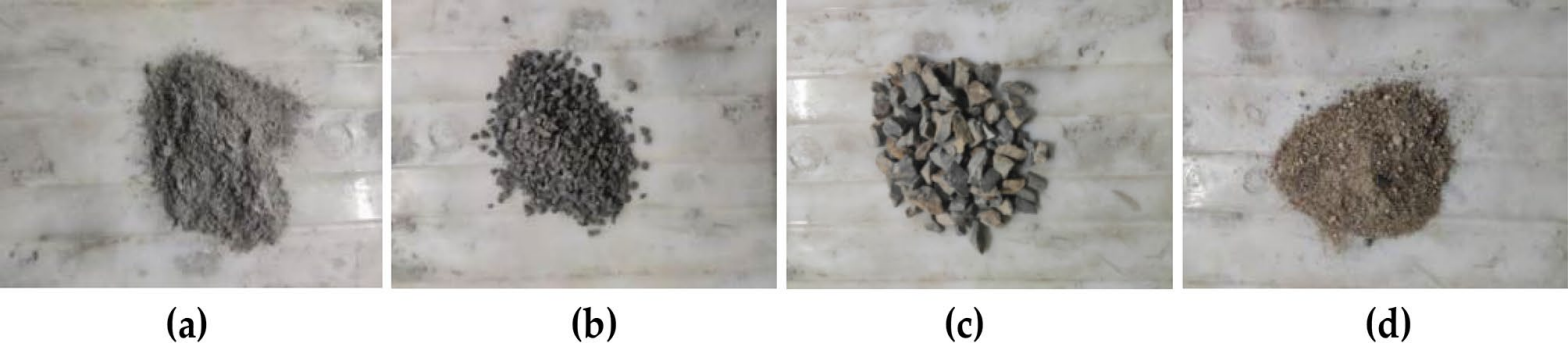
Concrete mix design materials: **(a)** cement; **(b)** 5–10 mm aggregate; **(c)** 10–19 mm aggregate; **(d)** river sand.

The FRP-confined concrete comprises originally plain concrete, an external FRP wrapping material, and an epoxy adhesive. Two types of external confined materials are selected: CFRP and GFRP, and the material property is shown in Table 1. Epoxy resins A and B are used as the adhesive (the volume mix ratio is 2:1). Figure 1 illustrates the detailed information on the concrete mix design material. Swan brand PO 42.5 Portland cement is used in Fig. 1a. The coarse aggregates are basalt crushed stone with diameters of 5-10 and 10-19 mm, respectively, as shown in Fig. 1b,c. Natural river sand with a fineness modulus of 2.3 is used as the fine aggregate in Fig. 1d. Sodium sulfate solution is prepared using anhydrous sodium sulfate granules and a specific amount of water.

**Table 1:** Reinforcing material performance index.

Types of reinforcement materials	Fiber specific gravity (g/cm^3^)	Design thickness (mm)	Tensile strength (MPa)	Tensile modulus of elasticity (MPa)	Elongation (%)
CFRP	1.8	0.167	3500	2.3 × 100,000	1.5
GFRP	2.5	0.18	2500	8.0 × 10,000	2.3

The concrete design strength is 30 MPa, which results in a slump of between 40 and 100 mm for newly mixed cement concrete. The concrete mix proportions are shown in Table 2, in which the ratio of coarse aggregate between 5 and 10 mm and 10–20 mm is 3:7. The environmental coupled effect is simulated by initially preparing a NaSO_4_ solution with a mass fraction of 10%, then pouring the solution into the freeze–thaw cycle box.

**Table 2:** Concrete mix ratio and grade.

Crushed stone/(kg·m^3^)	Medium sand/(kg·m^3^)	Cement/(kg·m^3^)	Water/(kg·m^3^)	Strength grade	Water–cement ratio
1169	635	387	209	C30	0.54

### Specimen preparation and FRP confinement treatment

Concrete mixtures are prepared in a 0.5 m^3^ forced mixer, and the entire batch of concrete is used to place the required specimens. First, the concrete ingredients are batched in Table 2, and the cement, sand, and coarse aggregate are premixed for three minutes. Then, evenly distribute the water and stir for 5 min. Next, concrete specimens are cast in cylindrical molds and compacted on a vibrating table (the molds are 10 cm in diameter by 20 cm in height).

After curing for 28 days, the specimens are wrapped with FRP materials. Three techniques for reinforced concrete columns are discussed in this study, which included entirely confined, semi-confined, and unconfined. Two types of CFRP and GFRP are used for the confined material. FRP fully confined concrete is wrapped FRP with 20 cm height by 39 cm length. The top and bottom of the FRP-confined concrete are not encapsulated by epoxy. As a newly suggested confined technique, the semi-confined test processes are described as follows.

(1) FRP is cut into strips with dimensions of 2 cm in height by 39 cm in length.

(2) A ruler is used to mark the concrete cylindrical surface to determine the location of the FRP strips, which are 2.5 cm apart from each other. Then wrap the tape around the concrete areas that do not require FRP.

(3) The concrete surface is sanded with sandpaper until smooth and wiped with alcohol cotton, and its surface is applied with an epoxy resin. Then stick the FRP strips on the concrete surface by hand and squeeze out the gap to make the FRP fully fit the concrete surface to avoid the generation of air bubbles. Finally, the FRP strips are glued to the concrete surface from top to bottom according to the marks made by the ruler.

(4) Check whether the concrete and FRP are separated after half an hour. If the FRP appears to slip or bulge, it should be corrected immediately. The molded specimens need to cure for 7 days to guarantee the hardened strength.

(5) After curing, the tape on the concrete surface is peeled off by a utility knife, and finally, the FRP semi-confined concrete column is obtained.

**Figure 2:**
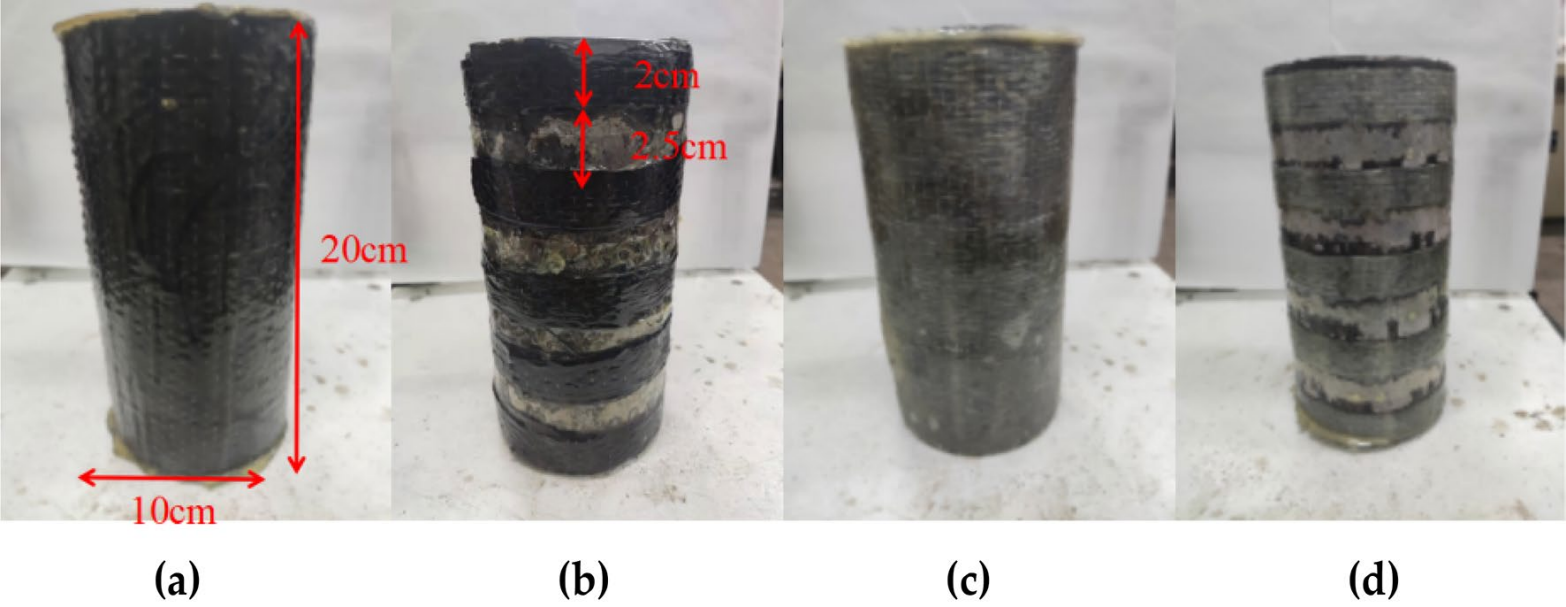
Confined style: **(a)** fully confined with CFRP; **(b)** semi-confined with CFRP; **(c)** fully confined with GFRP; **(d)** semi-confined with GFRP.

The results under different confinements are shown in Fig. 2. Figure 2a shows CFRP fully confined concrete, Fig. 2b shows CFRP semi-confined concrete, Fig. 2c shows GFRP fully confined concrete, and Fig. 2d shows GFRP semi-confined concrete.

### Various specimens under environmental conditions

There are four main parameters, and it aims to examine the influence of FRP-confined and erosion sequences on the anti-erosion performance of circular columns. Table 3 lists the number of concrete column specimens. Each category of specimens comprises three identical conditioned specimens to keep the data consistent. The average of three specimens analyzes all experimental results in this paper.

**Table 3:** Concrete column specimen number.

Pre-confined concrete	Freeze–thaw 0 times	Freeze–thaw 50 times	Freeze–thaw 100 times
CFRP fully confined	CF-FT00	CF-FT50	CF-FT100
CFRP semi-confined	CS-FT00	CS-FT50	CS-FT100
GFRP fully confined	GF-FT00	GF-FT50	GF-FT100
GFRP semi-confined	GS-FT00	GS-FT50	GS-FT100
Unconfined	U-FT00	U-FT50	U-FT100

Post-confined concrete	Freeze–thaw 0 time	Freeze–thaw 50 time	
CFRP fully confined after 50 freeze–thaw cycles	FT50-CF	FT50-CF-FT50	
CFRP semi-confined after 50 freeze–thaw cycles	FT50-CS	FT50-CS-FT50	
GFRP fully confined after 50 freeze–thaw cycles	FT50-GF	FT50-GF-FT50	
GFRP semi-confined after 50 freeze–thaw cycles	FT50-GS	FT50-GS-FT50	

‘G' means that the sample is made of GFRP material; ‘C' means that the sample is made of CFRP material; ‘U' means that the sample is unconfined; ‘F' means that the sample is fully confined; ‘S' means that the sample is semi-confined; the number after ‘FT' indicates the number of freeze–thaw cycles in sulfate solution. For example, ‘CS-FT50' means that the CFRP semi-confined concrete column is subjected to 50 freeze–thaw cycles in sulfate solution, and ‘FT50-CS-FT50' means that the unconfined concrete is first subjected to 50 freeze–thaw cycles, then CFRP semi-confined, and then 50 freeze–thaw cycles. And so on for other number meanings.

(1) Confined materials are classified as CFRP or GFRP. The impact of two fiber types on concrete reinforcing is compared.

(2) There are three confined techniques for concrete columns: entirely confined, semi-confined, and unconfined. The erosion resistance of semi-confined concrete columns is compared with the other two varieties.

(3) The erosion conditions are freeze–thaw cycles coupled with sulfate solution, and the freeze–thaw cycles are 0, 50, and 100 times, respectively. The effect of coupled erosion on FRP-confined concrete columns is investigated.

(4) The specimens are divided into three groups. The first group is FRP wrapped and then eroding, the second group is eroding first and then wrapped, and the third group is eroding first and then wrapped and then eroding again.

## Experimental programs

**Figure 3:**
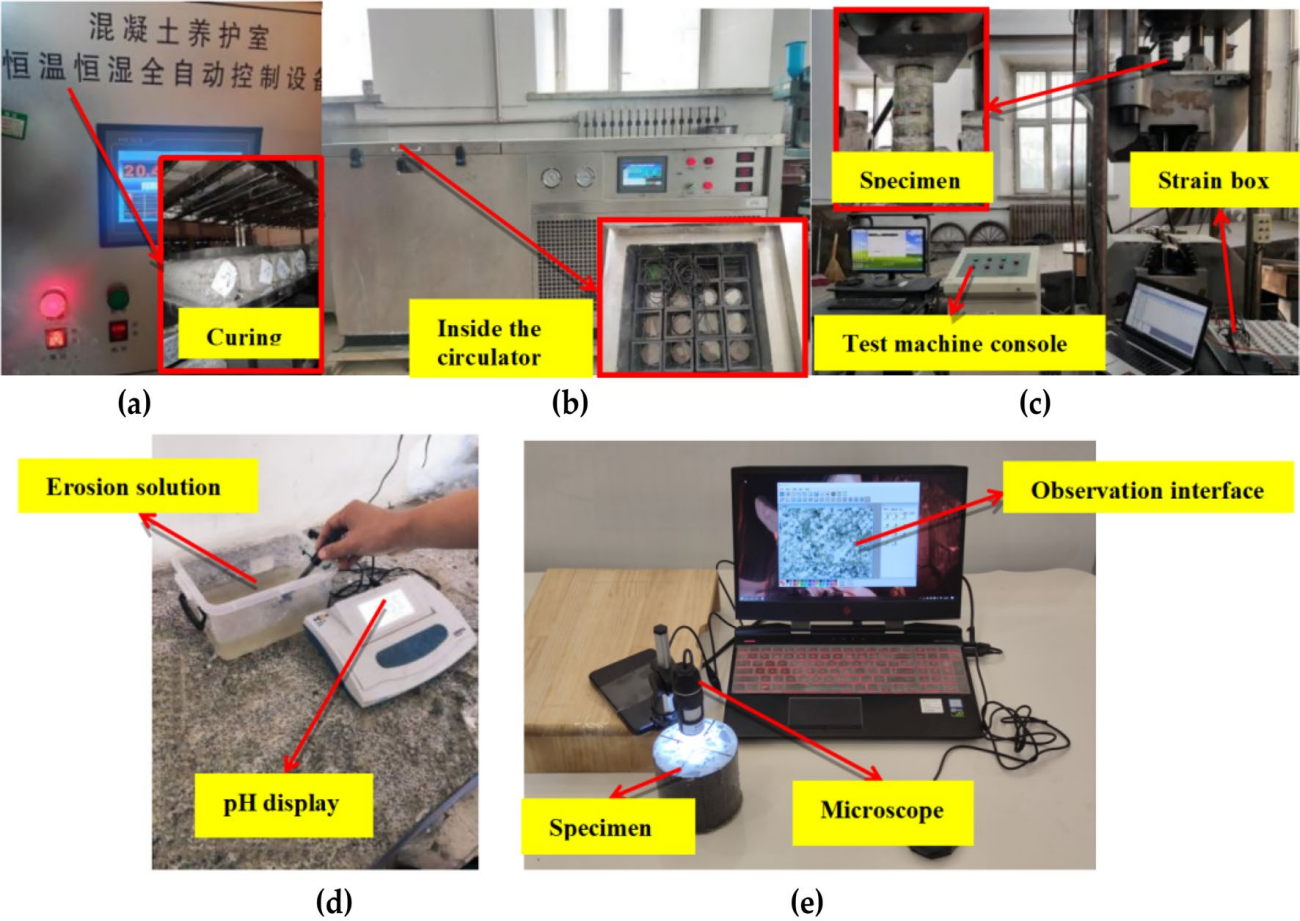
Test method: **(a)** constant temperature and humidity equipment; **(b)** freeze–thaw cycle machine; **(c)** universal test press; **(d)** pH tester; **(e)** microscopic observation.

The experiments programs are conducted using a universal testing machine, a tensile testing machine, a freeze–thaw cycle box (model CDR-Z), an electron microscope, a pH tester, a strain gauge, a displacement device, an SEM electron microscope, and an EDS energy spectrum analyzer in this study. The specimen is a concrete column of 10 cm in height by 20 cm in diameter. After being placed and compacted, the concrete will be cured for 28 days, as shown in Fig. 3a. All test specimens are demolded after casting and cured at 18–22 °C and 95% relative humidity for 28 days, and then some specimens are subjected to FRP wrapping.

### Freezing and thawing cycle tests

The freeze–thaw experiment used a rapid freezing method, as shown in Fig. 3b. According to GB/T 50082-2009 "Standard for Long-Term Performance and Durability Test of Ordinary Concrete," the concrete specimens are fully immersed in a 10% sodium sulfate solution at 15–20 °C for four days before freezing and thawing. After that, the sulfate attack coupled with freeze–thaw cycles begins and ends simultaneously. The duration of a freeze–thaw cycle is 2–4 h, and the thawing period should not be less than 1/4 of the cycle duration. The temperature at the sample's core should be kept between (−18±2) and (5±2) °C. The duration required to shift from frozen to the thawed state should not exceed ten minutes. Three cylindrical identity specimens for each category are utilized to investigate weight loss and solution pH changes for each 25 freeze–thaw cycle, as shown in Fig. 3d. After every 25 freeze–thaw cycles, the specimens are taken out and cleaned surface before determining their wet weight (W_d_). All experiments are performed on three sample replicates, and average values are used to discuss test results. The mass loss and strength loss formulas of the specimen are determined as follows:

1 $$\Delta {\text{W}}_{{\text{d}}} { = }\frac{{{\text{W}}_{{0}} - {\text{W}}_{{\text{d}}} }}{{{\text{W}}_{{0}} }}{{ \times 100}}$$

where ΔW_d_ is the weight loss of the specimen after every 25 freeze–thaw cycles (%), W_0_ is the average weight of the concrete specimen before the freeze–thaw cycle (kg), and W_d_ is the average weight of the concrete specimen after every 25 freeze–thaw cycles Weight (kg).

The strength degradation coefficient of the specimen is characterized by K_d_, and the formula is as follows:

2 $$\Delta {\text{K}}_{{\text{d}}} { = }\frac{{{\text{f}}_{{0}} - {\text{f}}_{{\text{d}}} }}{{{\text{f}}_{{0}} }}{{ \times 100}}$$

where ΔK_d_ is the strength loss rate (%) of the sample in every 50 freeze–thaw cycles, f_0_ is the average strength of the concrete sample before the freeze–thaw cycle (MPa), and f_d_ is the average strength of the concrete sample in every 50 freeze–thaw cycles (MPa).

### Mechanical property test

Figure 3c shows the concrete specimen compression test setup. According to the "Standard for Test Methods for Physical and Mechanical Properties of Concrete" (GBT50081-2019), the test method for the compressive strength test of concrete columns is determined. The compression test loading speed is 0.5 MPa/s, and continuous and consistent loading is used throughout the trial. The load–displacement relationship of each specimen is recorded during the mechanical test. Strain gauges are attached to the outer surfaces of the concrete and FRP layers of the specimens to measure the axial and horizontal strains. The strain box is used in the mechanical test to record the specimen's strain variation during the compression test.

### Analytical methods

A sample of the freeze–thaw solution is removed and placed in a container every 25 freeze–thaw cycles. Figure 3d shows the pH test of the solution sample in the container. Microscopic examination of the sample surface and cross-section under freeze–thaw conditions is shown in Fig. 3e. The surface conditions of different specimens after 50 and 100 freeze–thaw cycles in sulfate solution are observed by microscope. The microscope uses 400× magnification. The surface observation of the specimen mainly observed the erosion status of the FRP layer and the concrete outer layer. The section observation of the specimen mainly selects the erosion conditions at positions 5 mm, 10 mm, and 15 mm away from the outer layer. The formation products of sulfate and freeze–thaw cycle erosion require further verification. Therefore, the metamorphic surfaces of selected samples are examined with a scanning electron microscope (SEM) and equipped with an energy dispersive spectrometer (EDS).

### Test results and analysis

#### Surface erosion observation

**Figure 4:**
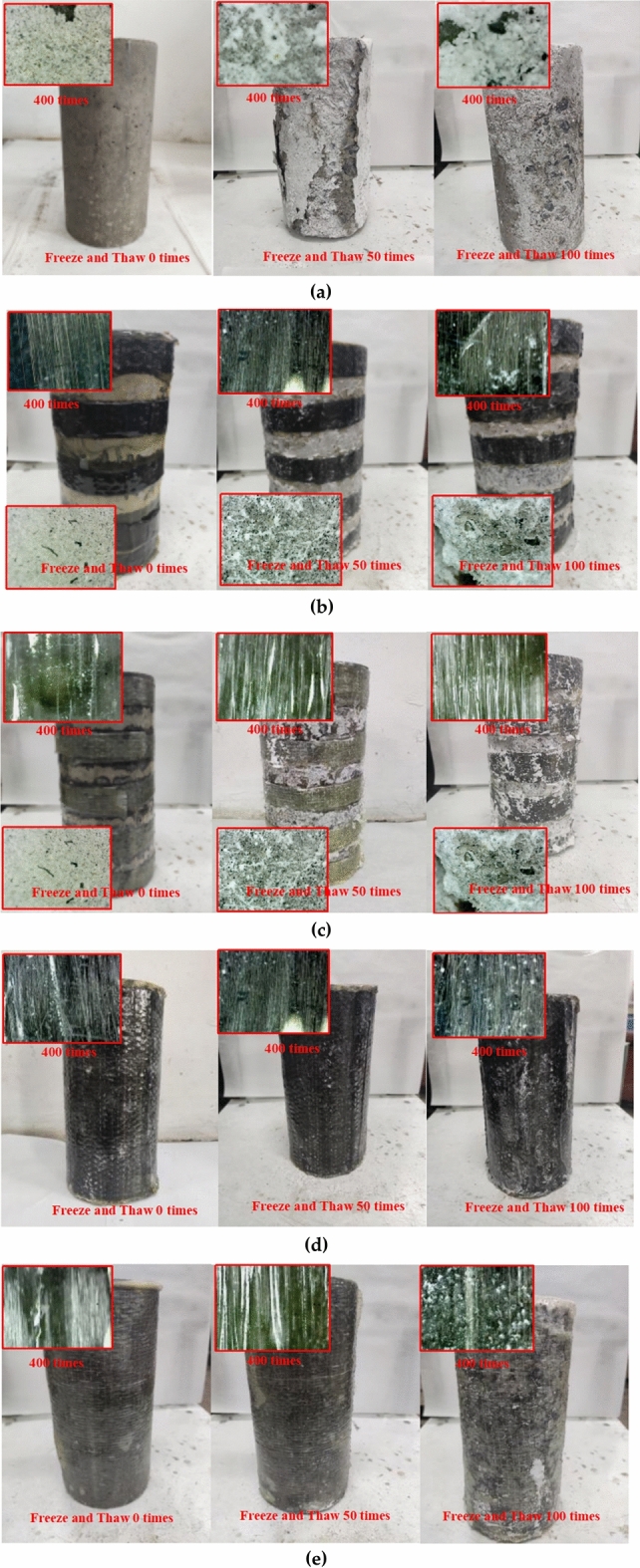
Freeze–thaw erosion appearance of concrete columns: **(a)** unconfined concrete column; **(b)** CFRP semi-confined concrete; **(c)** GFRP semi-confined concrete**; (d)** CFRP fully-confined concrete; **(e)** GFRP semi-confined concrete.

Visually examining the specimen's surface is performed using an electron microscope, and 400 magnification factors are selected. The surface damage degree of FRP semi-confined and unconfined concrete is quite severe when subjected to a freeze–thaw cycle coupled with sulfate erosion, whereas the surface damage degree of fully-confined concrete is negligible. As illustrated in Fig. 4a, the first category refers to the eroded appearance of unconfined concrete when subjected to a sodium sulfate coupled with freeze–thaw cycles from 0 to 100 times. The concrete specimen with no freeze–thaw erosion has a smooth surface devoid of visible features. After 50 times of erosion, the pulp block on the surface is partially peeled away, revealing a white pulp shell. After 100 times of erosion, visual inspection of the concrete surface revealed that the mortar shell had completely fallen off. Microscope observation reveals that the concrete surface eroded by 0 freeze–thaw cycles is smooth, and the aggregate and mortar on the surface are all on the same plane. An uneven rough surface is observed on the concrete surface eroded by 50 freeze–thaw cycles. It can be explained that partial mortar crumbles and a trace of white granular crystals adhere to the surface, mainly consisting of aggregates, mortar, and white crystals. After 100 freeze–thaw cycles, large areas of white crystals are found on the concrete surface, while the dark coarse aggregate is exposed to the external environment. At this point, the concrete surface is mainly composed of exposed aggregate and white crystals.

The second category is the corrosion appearance of CFRP and GFRP semi-confined concrete columns subjected to a freeze–thaw cycle coupled with sulfate erosion, as illustrated in Fig. 4b,c. Visual examination (1× magnification) revealed that the fiber layer's surface gradually developed some white powders that quickly dropped as the freeze–thaw cycles increased. The unconfined surface erosion of FRP semi-confined concrete becomes more pronounced as the number of freeze–thaw cycles increases. A ‘bulge' phenomenon is visible (the unconfined surface mortar of the concrete column is on the verge of collapsing). However, the spalling phenomenon is partially hampered by the nearby carbon fiber coating). Microscopically, the synthetic carbon fibers appear as white filaments on a black background at 400× magnification. They appear white due to the circular shape of the fibers and their exposure to uneven light, but the carbon fiber strands themselves are black. The glass fiber is initially white and filamentous, but when exposed to the adhesive, it becomes transparent, allowing for a clear view of the state of the concrete within the glass fiber cloth. The fiberglass is a bright white color, while the adhesive is a yellowish hue. Both are light in color, so the adhesive's color will obscure the fiberglass strands, giving the overall appearance of a yellowish tint. The carbon and glass fibers are protected with exterior epoxy resin, and no damage occurs. More voids and a few white crystals on the surface became visible as the number of freeze–thaw erosions increased. With the increasing sulfate-freeze cycles, the adhesive gradually thins, the pale yellow fades, and the fibers become visible.

The third category is the eroded appearance of CFRP and GFRP fully confined concrete subjected to a freeze–thaw cycle coupled with sulfate erosion, as illustrated in Fig. 4d,e. Again, the observations are similar to the second type of concrete column confinement section results.

Contrast the phenomena observed following the application of the three confinement techniques described above. The fiber fabric in FRP fully confined concrete remained stable as the freeze–thaw cycles increased. On the other hand, the adhesive ring layer is thinner on the surface. Epoxy resin mainly reacts with active hydrogen ions in sulfuric acid to open the ring and hardly reacts with sulfate^28^. Therefore, it can be considered that erosion mainly changes the enhancement effect of FRP by changing the properties of the adhesive layer through freeze–thaw cycles. The concrete surface of FRP semi-confined concrete has the same erosion phenomenon as the unconfined concrete surface. Its FRP layer is consistent with the FRP layer of fully confined concrete, and the damage is not apparent. However, extensive erosion cracks occur at the intersection of fiber strips and exposed concrete in FRP semi-confined concrete. The erosion of the unconfined concrete surface becomes more severe as the number of freeze–thaw cycles increases.

### Internal erosion observation

**Figure 5:**
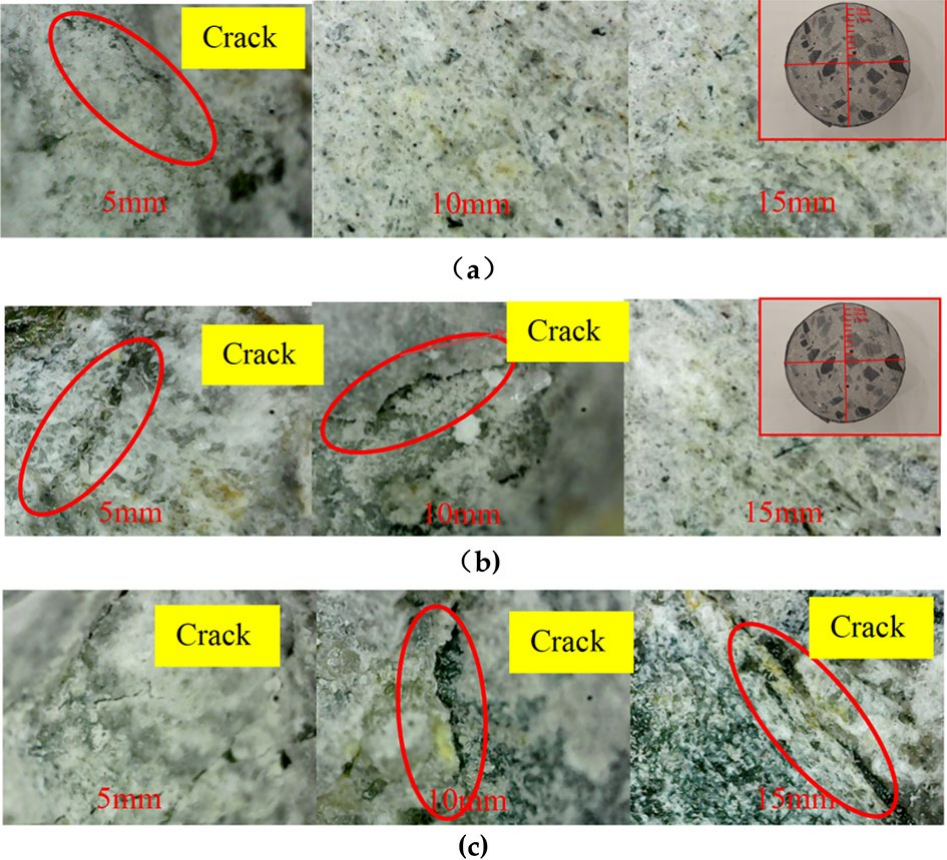
Internal microscope observation of concrete column section: **(a)** FRP fully confined; **(b)** FRP semi-confined; **(c)** unconfined.

The interiors of FRP fully-confined, semi-confined, and unconfined concrete exhibit significant differences when eroded by freeze–thaw cycles coupled with sulfate solutions. Transverse cuts are made in the test pieces, and the cross-sections are observed using a 400× magnification electron microscope. Figure 5 shows the microscope images of 5 mm, 10 mm, and 15 mm from the contact surface of concrete and solution, respectively. It is observed that the concrete damage is gradually eroded from the surface to the interior when a sodium sulfate solution is coupled with freeze–thaw action. Because the internal erosion conditions of CFRP and GFRP-confined concrete are identical, this section will not compare the two types of confinement materials.

Internal erosion of the FRP fully confined concrete is depicted in Fig. 5a. At a distance of 5 mm, cracks are visible, the surface is relatively smooth, and no crystals have precipitated. The surface is smooth and crystal-free, between 10 and 15 mm. Internal erosion of the FRP semi-confined concrete is depicted in Fig. 5b. Cracks and white crystals are visible at 5 mm and 10 mm, while at 15 mm, the surface is smooth. Figure 5c shows the section of the FRP-confined concrete column with cracks found at 5, 10, and 15 mm. A few white crystals in the cracks become increasingly rare as they move from the concrete's exterior to its interior. Unconfined concrete columns experience the most severe erosion, followed by FRP semi-confined concrete columns. Within 100 freeze–thaw cycles, sodium sulfate had little effect on the interior of the FRP fully restrained concrete specimen. This indicates that the main reason for FRP fully confined concrete erosion is freeze–thaw within a certain period of coupled erosion. Sectional observation revealed that the section immediately preceding freezing and thawing is smooth and aggregate-free. After the concrete has been frozen and thawed, the cracks are visible, as is the aggregate, and the cracks are densely packed with white granular crystals. Studies^27^ have shown that when concrete is placed in a sodium sulfate solution, sodium sulfate will penetrate the concrete, part of it will precipitate in the form of sodium sulfate crystals, and part of it will react with the cement. Sodium sulfate crystals and reaction products appear as white granules.

The concrete under FRP full confinement produces cracks under coupled erosion, but the section is smooth and crystal-free. On the other hand, FRP semi-confined and unconfined concrete sections develop internal cracks and crystals under coupled erosion. According to the picture description and previous research^29^, the coupling erosion process of unconfined and FRP semi-confined concrete is divided into two stages. The first stage of concrete cracks is due to freeze–thaw expansion and contraction. As the sulfate penetrates the concrete and becomes visible, the appropriate sulfate fills the cracks created by freeze–thaw and shrinkage from hydration reactions. Therefore, sulfate has a particularly protective effect on concrete in the initial stage and can improve the mechanical properties of concrete to a certain extent. The second stage of sulfate erosion continues, entering cracks or voids and reacting with cement to form alum. As a result, the fracture volume expands and causes damage. At this time, the expansion and contraction reactions related to freezing and thawing will aggravate the internal damage of the concrete, resulting in a decrease in the bearing capacity.

### Analysis of pH change

**Figure 6:**
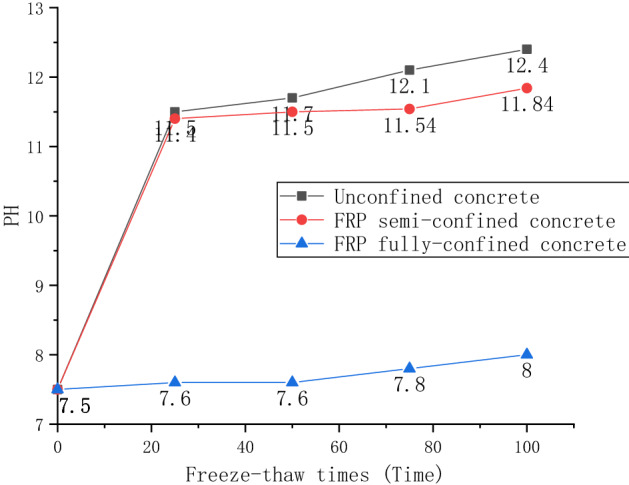
pH change.

Figure 6 shows the pH changes of concrete immersion solutions of three confined techniques monitored after 0, 25, 50, 75, and 100 freeze–thaw cycles. Unconfined and FRP semi-confined concrete solutions showed the fastest pH rise in 0 to 25 freeze–thaw cycles. Their pH increased from 7.5 to 11.5 and 11.4, respectively. With the increase in the number of freeze–thaw cycles, the pH increase gradually slowed down within 25–100 freeze–thaw cycles. Their pH increased from 11.5 and 11.4 to 12.4 and 11.84, respectively. Since the FRP fully restrained concrete wraps the FRP layer, the sodium sulfate solution is difficult to penetrate. At the same time, it is difficult for the cement composition to penetrate the external solution. Therefore, the pH gradually increased from 7.5 to 8.0 within 0 to 100 freeze–thaw cycles. The reasons for pH changes are analyzed as follows. The silicate in the concrete combines with the hydrogen ions in the water to form silicic acid, and the remaining OH^−^ causes the pH of the saturated solution to increase. The pH changes are more significant in 0–25 freeze–thaw cycles, and the changes are not apparent in 25–100 freeze–thaw cycles^30^. However, it is found here that the pH continued to increase over 25–100 freeze–thaw cycles. It can be explained that sodium sulfate reacts chemically with the interior of the concrete, changing the pH of the solution. Chemical composition analysis shows that the following reactions occur between concrete and sodium sulfate.

3 $${\text{3(CaSO}}_{{4}} \cdot {\text{2H}}_{{2}} {\text{O) + 4CaO}} \cdot {\text{Al}}_{{2}} {\text{O}}_{{3}} \cdot {\text{13H}}_{{2}} {\text{O + 14H}}_{{2}} {\text{O = 3CaO}} \cdot {\text{Al}}_{{2}} {\text{O}} \cdot {\text{CaSO}}_{{4}} \cdot {\text{32H2O + Ca(OH)}}_{{2}} \,$$

4 $${\text{Ca(OH)}}_{{2}} {\text{ + Na}}_{{2}} {\text{SO}}_{{4}} {\text{ + 2H}}_{{2}} {\text{O = CaSO}}_{{4}} \cdot {\text{2H}}_{{2}} {\text{O + 2NaOH }}$$

According to formulas (3) and (4), it can be seen that sodium sulfate and calcium hydroxide in cement generate gypsum (calcium sulfate), and calcium sulfate further reacts with calcium meta aluminate in cement to form alum crystals. The reaction (4) is accompanied by the formation of alkaline OH^**-**^, so the pH increases. Furthermore, because the reaction is reversible, the pH increases at a particular time and changes slowly.

### Mass change analysis

**Figure 7:**
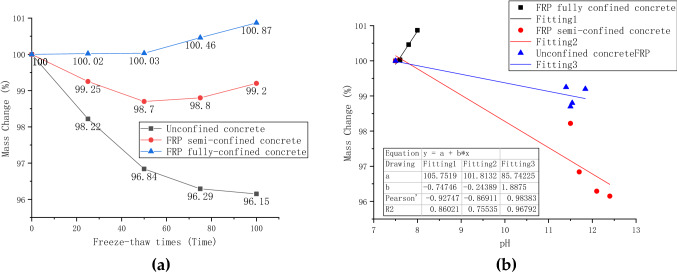
Mass change: **(a)** the relationship between mass change and the number of freeze–thaw cycles; **(b)** the relationship between mass change and pH.

The mass loss of FRP fully-confined, semi-confined, and unconfined concrete during freeze−thaw cycles in sulfate solution is shown in Fig. 7a. The most apparent change in mass loss is for unconfined concrete. Unconfined concrete lost about 3.2% mass after 50 freeze–thaw attacks and about 3.85% after 100 freeze–thaw attacks. It shows that as the number of freeze–thaw increases, the effect of coupled erosion on the mass of unconfined concrete decreases. However, by observing the surface of the specimen, it is found that the loss of mortar after 100 freeze–thaw cycles is more severe than that after 50 cycles of freeze–thaw cycles. Combined with the research in the previous section, it can be speculated that the penetration of sulfate into the concrete leads to the slowing of mass loss. At the same time, it can be predicted from chemical equations (3) and (4) that alum and gypsum generated internally will also lead to a slower mass loss.

The change of mass loss of FRP semi-confined concrete first decreased and then increased. After 50 freeze–thaw erosions, the mass loss of FRP semi-confined concrete is about 1.3%. The mass loss after 100 cycles is 0.8%. Therefore, it can be concluded that sodium sulfate penetrates the concrete in unconfined concrete. In addition, observation of the surface of the specimens also found that the fiber strips can resist mortar spalling in unconfined areas, thereby reducing mass loss.

The change in mass loss of FRP fully confined concrete is different from the former two. It does not lose mass but gains mass. After 50 freeze–thaw erosions, the mass increased by about 0.08%. After 100 times, its mass increased by about 0.428%. Because the concrete is completely encased, the mortar on the concrete surface will not fall off, resulting in virtually any loss of mass. On the other hand, the penetration of water and sulfate from the high content surface into the low content of interior concrete can also increase concrete mass.

There are few previous studies on the relationship between pH and mass loss of FRP-confined concrete under erosive conditions. Most studies mainly discuss the relationship between mass loss, elastic modulus, and strength loss. Figure 7b shows the relationship between concrete pH and mass loss under three constraints. A predictive model is presented to predict the mass loss of concrete with three confined techniques at different pH values. It can be seen from the Fig. 7b that the Pearson coefficient is high, indicating that there is indeed a correlation between pH and mass loss. The r-squared for unconfined concrete, semi-confined concrete, and fully confined concrete are 0.86, 0.75, and 0.96, respectively. This indicates that the pH change and mass loss of fully confined concrete under the coupled conditions of sulfate and freeze–thaw are relatively linear. The pH gradually increased with the chemical reaction of cement and aqueous solution in unconfined concrete and FRP semi-confined concrete. As a result, the concrete surface was gradually eroded and dropped, leading to weight loss. On the other hand, the pH variation of fully confined concrete is slight because the FRP layer slows the chemical reaction of the cement with the aqueous solution. Therefore, no visible surface erode was observed for fully confined concrete, but it gained weight due to the saturation effect of the absorbing sulfate solution.

### SEM and EDS analysis

**Figure 8:**
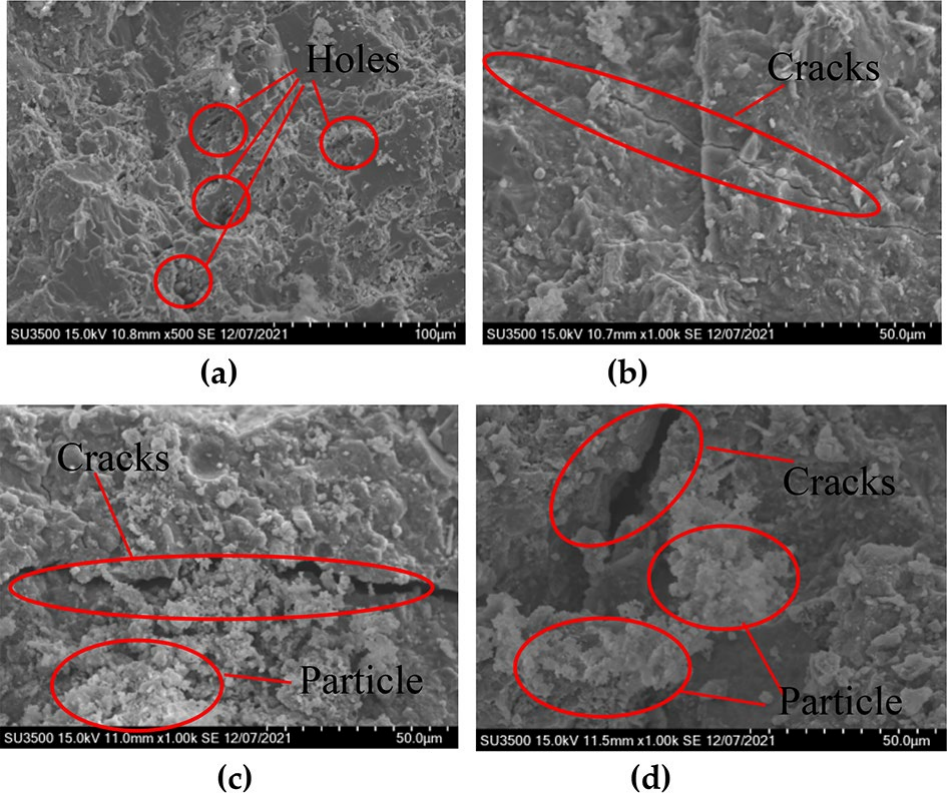
Electron microscope scans of specimen before and after erosion: **(a)** unconfined concrete of before erosion; **(b)** after erosio of FRP fully confined; **(c)** after erosion of FRP semi-confined concrete; **(d)** after erosion of unconfined concrete.

**Figure 9:**
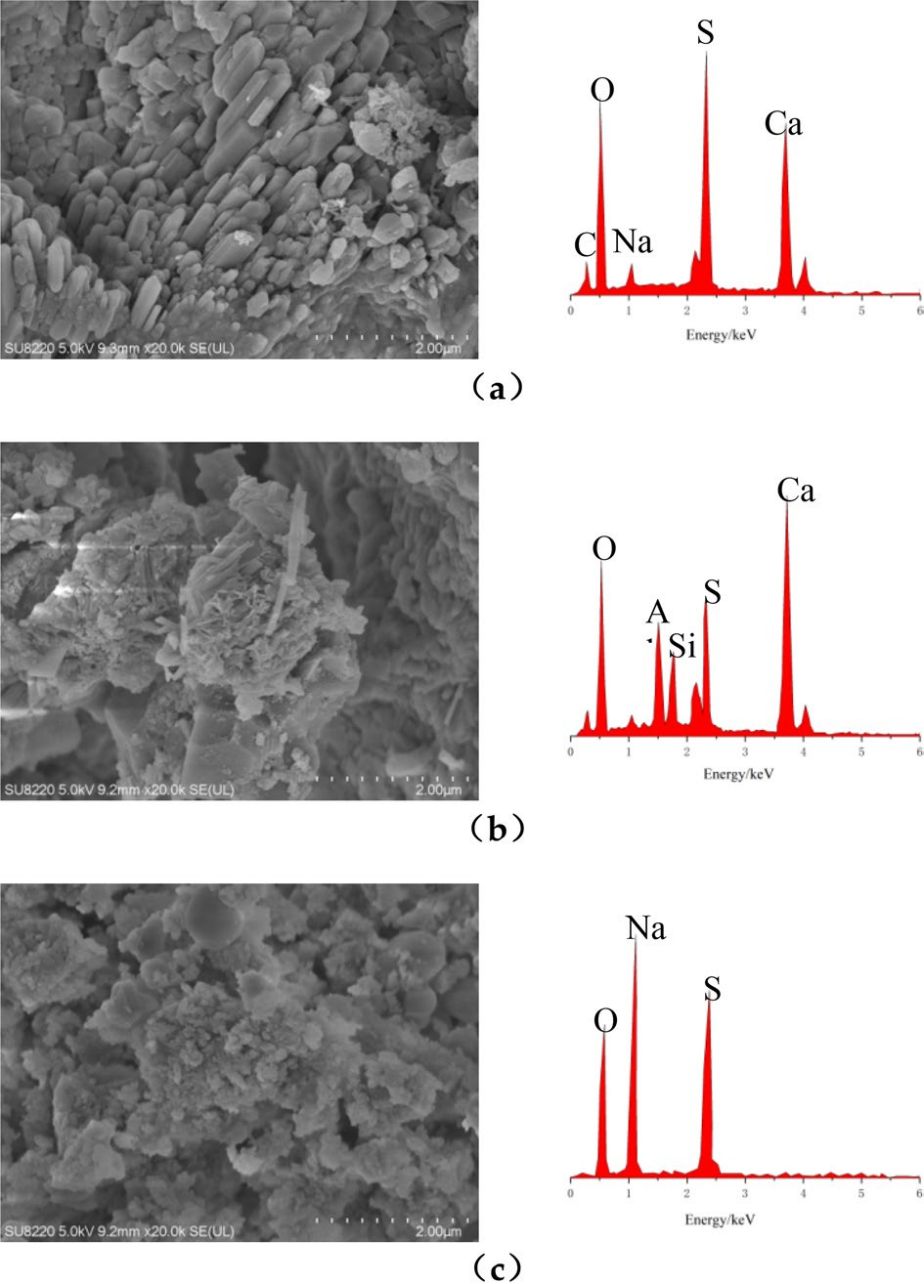
EDS analysis: **(a)** gypsum (calcium sulfate); **(b)** alum; **(c)** mirabilite (sodium sulfate crystal).

Figure 8 shows the results of SEM scanning of the sodium sulfate freeze–thaw erosion sample. Electron microscopy examined samples collected from blocks taken from the outer layers of concrete columns. Figure 8a is the scanning electron microscope image of unconfined concrete before being eroded. It is observed that there are many holes on the surface of the sample, which affects the strength of the concrete column itself before freeze–thaw erosion. Figure 8b shows the electron microscope scan of the FRP fully confined concrete specimen after 100 freeze–thaw erosions. Cracks caused by freezing and thawing in the specimen can be detected. However, the surface is relatively smooth, and no crystals are present. Therefore, unfilled cracks are more visible. Figure 8c shows the FRP semi-confined concrete specimen after 100 freeze–thaw erosions. It is clear that the cracks have widened, and some particles have formed between them. Part of these particles is attached to the crack. The SEM scan of the sampling of the unconfined concrete column is shown in Fig. 8d, and the phenomenon is consistent with the semi-confinement. To further elucidate the composition of the particles, the particles at the crack are further enlarged and analyzed using EDS spectroscopy. Particles mainly come in three different shapes. According to the energy spectrum analysis, the first category is shown in Fig. 9a, which is a regular bulk crystal, mainly composed of O, S, Ca, and other elements. It can be determined that the material is mainly composed of gypsum (calcium sulfate) when combining the previous formulas (3) and (4). The second type is shown in Fig. 9b; it is a needle-shaped non-directional object through energy spectrum analysis, mainly composed of O, Al, S, and Ca. The combined formula shows that the material is mainly composed of alum. The third type is shown in Fig. 9c, which is an irregular bulk, determined by energy spectrum analysis, mainly composed of O, Na, and S components. It turns out that these are mainly sodium sulfate crystals. Scanning electron microscopy revealed that most voids are filled with sodium sulfate crystals, as shown in Fig. 9c, along with a small amount of gypsum and alum.

The following conclusions are obtained from the analysis. The electron microscope images of the three specimens are all 1k ×, and the cracks and erosion products in the images are found and observed. Unconfined concrete has the widest cracks and contains many particles. FRP semi-confined is inferior to unconfined concrete in crack width and particle count. FRP fully-confined concrete crack width after freeze–thaw coupled erosion is the smallest and free of particles. All these indicate that the FRP fully confined concrete is least affected by the coupled erosion of freeze–thaw. The chemical processes inside the FRP semi-confined and unconfined concrete columns lead to the formation of alum and gypsum, and the infiltration of sulfates affects the pores. Although freeze–thaw cycles are the primary cause of concrete cracking, sulfate and its products fill some of the cracks and pores in the first place. However, as the number and time of erosion increased, the cracks continued to grow, and the volume of alum produced increased, squeezing the cracks. Ultimately, freeze–thaw and sulfate attack can reduce the strength of the column.

### Research on the frost resistance of FRP-confined concrete

**Figure 10:**
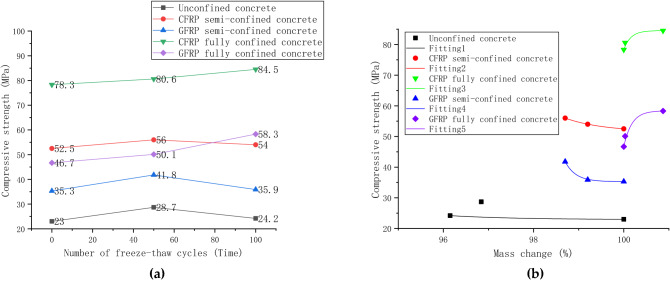
Comparison of different confined technologies and material changes: (**a**) variation of concrete strength with different confinement techniques; (**b**) the relationship between mass change and strength.

Axial compressive strength tests investigated the mechanical properties of two confinement materials and technologies subjected to freeze–thaw cycle erosion in a sodium sulfate solution. Figure 10a compares the compressive strength of concrete using different confined techniques and materials under erosive conditions and investigates the variation of its compressive strength with the number of freeze–thaw cycles. The strength of FRP semi-confined and unconfined concrete increased initially and then decreased slightly. The fully confined concrete exhibits a gradual increase in strength.

After 50 freeze–thaw cycles, the strength of CFRP fully confined concrete increases by 2.3 MPa, and the strength of semi-confined concrete increases by 3.5 MPa. The strength of GFRP fully confined 3.4 MPa increases concrete, and similarly, 6.5 MPa increases for GFRP semi-confined concrete. Studies have shown that cracks are generated inside FRP-confined concrete, and its strength will decrease under a single freeze–thaw condition^22^. However, the strength of the test results increased. It can be explained that the increase in strength is due to the addition of sodium sulfate to fill the pores in the concrete and cracks caused by freeze–thaw when considering the result of electron microscopy, mass loss, and pH change. After 100 freeze–thaw cycles, the strength of CFRP fully-confined concrete increased by 3.9 MPa, and the strength of semi-confined concrete decreased by 2 MPa. GFRP fully confined concrete increased by 8.2 MPa, while GFRP semi-confined concrete decreased by 5.9 MPa. This is because the freeze–thaw cycles cause the cracks to get progressively worse. The excessive volume of alum and gypsum generated by the reaction of sodium sulfate with cement will also squeeze cracks and aggravate internal damage. The strength of unconfined concrete increased by 5.7 MPa after 50 freeze–thaw cycles and decreased by 4.5 MPa after 100 freeze–thaw cycles. The variation law of strength is similar to that of FRP semi-confined, which increases first and then decreases.

The strength variation of CFRP and GFRP-confined concrete under freeze–thaw cycles can also be compared in Fig. 10a. After 50 and 100 freeze–thaw cycles, the strength of CFRP-confined concrete is significantly higher than that of GFRP-confined concrete, regardless of whether it was fully confined or semi-confined concrete. In addition, it can be seen that the fully-confined technique can amplify the strength gap caused by different materials more than the semi-confined technique by comparing the strength changes of the two materials.

Mass loss is an essential indicator for evaluating the frost resistance of concrete in the freeze–thaw test. Unconfined concrete mass loss increases continuously under a single freeze–thaw condition^22^. However, under the action of freeze–thaw cycles in sodium sulfate solution, the mass loss of concrete with different confined methods and materials is not consistent. Therefore, this paper analyzes the correlation between the mass loss and the sample's compressive strength and uses the Origin software to fit the curve in Fig. 10b. The strength prediction models of different specimens based on mass loss are obtained, and the relevant results and parameters are shown in Table 4. In this prediction model, the fitting degree R^2^ of the unconfined concrete is small, and other prediction formulas have a high fitting degree.

**Table 4:** Predictive model and parameters.

Equation	y = a − b × c^x^				
Curve fitting	1	2	3	4	5
a	22.9006	51.51439	84.52282	35.27363	58.38671
b	−9.85E + 27	−4.15E + 50	5.66E + 280	−3.53E + 182	7.16E + 245
c	0.51291	0.31171	0.00159	0.01441	0.00357
R^2^	−0.37304	0.99994	0.926	0.99904	0.96481

### Differences in freeze–thaw performance of pre-confined and post-confined concrete

**Figure 11:**
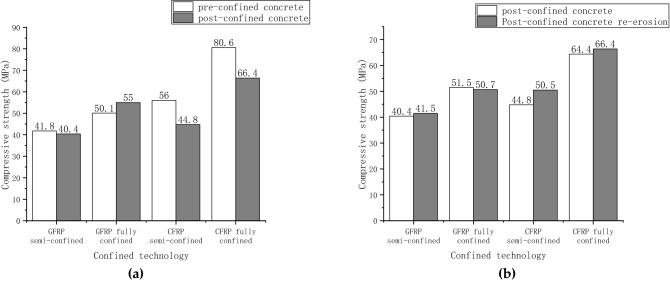
Influence of confined sequence on concrete: **(a)** strength comparison of pre-confined and post-confined concrete; **(b)** strength comparison of post-confined and re-coupling concrete.

This study aimed to determine the effect of confinement on the operational performance of concrete eroded by freeze–thaw cycles in a sulfate solution. In this context, the term ‘working performance' refers primarily to the eroded concrete's ability to regain its strength following confinement and its ability to resist further erosion following confinement. Erosion conditions are 50 freeze–thaw cycles in 10% sodium sulfate solution. Figure 11 shows the strength of concrete in the order of confinement and erosion for different confinement materials and techniques. Pre-confined concrete means that the ordinary concrete is first confined by FRP and then eroded. Post-confined concrete means that the ordinary concrete is eroded first and then confined.

Figure 11a compares the CFRP and GFRP strength relationship between pre-confined and post-confined concrete. The strength of the post-confined GFRP semi-confined concrete is 96% of the pre-confined concrete. The post-confined GFRP fully confined concrete strength is 109% that of the pre-confined concrete. The strength of the post-confined CFRP semi-confined concrete is approximately 80% of that of the pre-confined concrete. Then, the GFRP fully confined concrete with post-confined strength reaches 82.3% of the pre-confined strength. Concrete increases in strength after 50 freeze–thaw cycles due to sulfate infiltration. It is suggested that the confined concrete after coupled erosion should be higher than the uneroded but confined concrete. However, the strength of post-restrained concrete is lower than that of pre-confined concrete. Concrete surface erosion analysis showed that the surface of the pre-confined concrete is smooth, whereas the surface of the post-confined concrete is rough and dotted with powder crystals. At this point, the use of fiber layers to confine the concrete surface results in uneven confinement and concentrated stress when the specimen's axial center is compressed. Therefore, it can be observed that the post-confined concrete strength is less than that of the pre-confined concrete. Additionally, the test demonstrates that when concrete is eroded in engineering practice, the surface becomes rough and uneven, reducing the concrete's repairing ability.

Figure 11b shows the strength of post-confined concrete and its re-coupled erosion. The re-coupling erosion condition is still 50 freeze–thaw cycles in 10% sodium sulfate solution. The original strength represents the strength of the post-confined concrete before re-erosion. After re-erosion, the CFRP semi-confined concrete can reach 102% of its original strength, while the GFRP fully-confined concrete can re-erode to 98% of its original strength.The CFRP semi-confined concrete can reach 112% of its original strength, while the GFRP fully-confined concrete can re-erode to 103% of its original strength. The test results show that the post-confined concrete can still maintain a high compressive strength after being eroded again. It is also shown that the confinement material and confinement technique have insignificant effects on the order of confinement and coupled erosion.

### Curves of stress–strain for various confined materials and techniques

Stress–strain models for concrete with different confined materials and techniques are investigated under coupled erosion conditions. Firstly, the stress–strain law of different concretes under the coupled action of sulfate and freeze–thaw is analyzed. Secondly, the applicability of the previous ultimate stress–strain prediction models is discussed, and the ultimate stress–strain models of CFRP and GFRP materials suitable for the coupled erosion conditions of sulfate and freeze–thaw cycles are established.

### Stress–strain curve analysis of unconfined concrete

**Figure 12:**
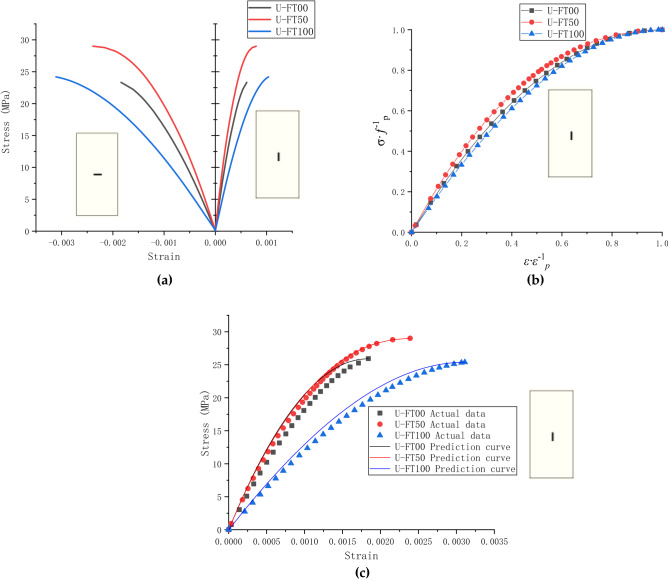
Unconfined concrete stress–strain curve: **(a)** measured stress–strain curve ; **(b)** Guo Zhenhai's dimensionless stress–strain curve; **(c)** measured and fitted stress–strain curves.

The stress–strain curves for unconfined concrete after 0, 50, and 100 freeze–thaw cycles in sulfate solution are depicted in Fig. 12a. Guo's constitutive equation^31^ of concrete under uniaxial compression is cited as shown in Eq. (5). It is used to fit the stress–strain curve and determine the value of ‘a'. As a result of the final calculation, ‘a' equals 2.0, 2.3, and 1.8.

5 $${\text{y = ax + (3}} - {\text{2a) x}}^{{2}} {\text{ + (a}} - {\text{2) x}}^{{3}} ,\quad {\text{0 < x < 1 }}$$

According to Guo's model, the smaller value of parameter ‘a', the narrower curve, and the smaller integral area are observed. It can be explained that it demonstrates ‘a' decrease in ductility and plastic deformation capability. On the other hand, the larger the parameter ‘a', the more significant the ability. Thus, parameter ‘a' can be used to compare the mechanical properties of concrete. Figure 12b shows the dimensionless stress–strain fitting curves of concrete in sulfate solution for different freeze–thaw times. The size of the integral area under the dimensionless stress–strain curve can be clearly observed. Careful observation of the critical area and rising speed of the dimensionless stress–strain curve shows the influence of a's value on it. The smaller the ‘a', the steeper the strain rise, indicating a decrease in ductility. The more significant the ‘a', the more gradual the rise in strain, explaining the increase in ductility. However, the ductility here only represents the ductility of the dimensionless stress–strain curve. After 50 freeze–thaw cycles, the concrete exhibited good ductility and plasticity. On the other hand, after 100 freeze–thaw cycles, the value of parameter a decreases, resulting in a decrease in overall area and ductility. The stress–strain curve predicted by the Guo's model and the measured data are shown in Figure 12c. The predicted curve is in good agreement with the measured data.

Figure 12c shows the stress–strain relationship before and after coupled erosion. After 50 freeze–thaw cycles, the ultimate strain and stress also increased due to the increased compaction of the concrete and the infiltration of sodium sulfate, which indicates that concrete's ductility and compressive strength improve during the initial coupled erosion stage. At the same stress level, the axial strain of the FT100 specimen is much higher than that of the FT50 specimen. Because for concrete with a compressive strength of 30 MPa, the yield stress occurs around 9–10 MPa. Therefore, ductility increases with the number of freeze–thaw cycles. In addition, as the number of freeze–thaw cycles continues to increase, the expansion of concrete cracks increases. At this time, the effect of freezing and thawing leads to the internal loosening of the concrete, decreases the strength, and increases the ductility.

### Stress–strain curve analysis of FRP-confined concrete

**Figure 13:**
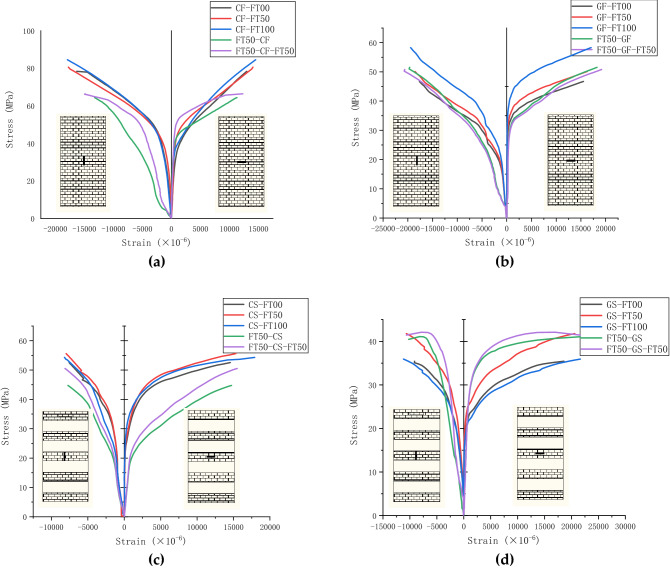
Stress–strain curves of concrete with different confined techniques under coupled erosion**: (a)** stress–strain curve of CFRP fully-confined concrete; **(b)** stress–strain curve of GFRP fully-confined concrete ; **(c)** stress–strain curve of CFRP semi-confined concrete; **(d)** stress–strain curve of GFRP semi-confined concrete.

The stress–strain curves of various confined materials and technologies during freeze–thaw cycles in sodium sulfate solution are shown in Fig. 13. The longitudinal strain is depicted on the left, while the transverse strain is depicted on the right. Three stages can be identified in the stress–strain curve of FRP-confined concrete. The axial strain of FRP semi-confined and fully confined concrete is comparable to that of unconfined concrete in the first stage. This demonstrates that the fiber layer does not exert its constraining effect at this stage. The lateral strain of the confined concrete changes very little, and it is readily apparent that the axial strain is greater than the lateral strain. As the stress level continues to rise, the second stage begins. At this point, the curve becomes gentler, and the curvature increases gradually. Transverse strain changes at a much faster rate than longitudinal strain are observed. The concrete's interior is gradually destroyed, the fiber layer gradually becomes involved, and both the concrete and the fibers bear the axial stress simultaneously. When the stress is increased further, the concrete's interior rapidly deteriorates and enters the third stage. This stage is primarily concerned with constraining the fiber layer to bear the specimen's axial stress. At this point, linear changes are resumed. The transverse strain is significantly increased. The longitudinal strain changes at a slightly slower rate than the transverse strain, and it can be clearly observed that the slope of the transverse strain is smaller than that of the longitudinal strain). The concrete fails when the fiber layer's ultimate tensile strain is reached, signaling the end of the third stage.

The stress–strain curves of CFRP and GFRP fully confined concrete columns under freeze–thaw conditions are shown in Fig. 13a,b. In the first stage, the strain change of the eroded and then confined concrete is more significant than that of the unconfined concrete, and the stress is always lower. The curve of FRP fully confined concrete that has been eroded and then confined and eroded again is not significantly different from the curve of FRP fully confined concrete that has not been eroded, indicating that fully confined concrete has been eroded then confined can still maintain good erosion resistance. There is no significant difference in the first stage of stress–strain change as the number of erosion increases, and even in GFRP, the number of erosion is slightly higher. Consider the semi-confined concrete of CFRP and GFRP in Fig. 13b,d. FRP semi-confined concrete has a lower resistance to erosion than FRP fully-confined concrete, but the change rule is the same.

Figure 13a,c show the stress–strain curves of CFRP fully-confined and semi-confined concrete, respectively. The longitudinal strain curve of CFRP semi-confined concrete is steeper than that of CFRP fully confined concrete. This is because the fiber cloth strips in the CFRP semi-confined concrete are separated. Because the confinement force of the fiber strips of CFRP semi-confined concrete is less than that of fully confined concrete. Therefore, the longitudinal strain of semi-confined concrete changes faster in axial compression, and the ultimate strain becomes smaller. However, the ultimate lateral strain is only affected by the material of the FRP itself, so there is no change. Figure 13c stress–strain curve shows that the lateral strain of CFRP semi-confined coagulation is very short in the first and second stages and develops rapidly, and the slope is relatively stable in the third stage. It can be explained that the confinement area of CFRP semi-confined concrete is smaller, which is half that of fully confined concrete. As a result, the concrete quickly loses its working ability. It is forced to rely on the lateral restraint force of the fiber layer to reduce the deformation of the specimen. Therefore, the halving of the fiber layer area accelerates the change in strain until failure. The phenomenon of CFRP-confined concrete can also be obtained by comparing the GFRP fully-confined and semi-confined concrete in Fig. 15b,d.

Table 5 shows the ultimate stress and strain of FRP-confined concrete and unconfined concrete. The ε′_cc_/ε′_co_ is defined with the extension ratio, which can indicate the degree of improvement of the deformation capacity of FRP-confined concrete compared with unconfined concrete. Similarly, the f′_cc_/f′_co_ is defined with the enhancement ratio, indicating the improvement of the axial compressive capacity of FRP-confined concrete compared with unconfined ordinary concrete. The observation table shows that CFRP fully confined concrete's confined ratio is the largest, followed by GFRP fully confined concrete, CFRP semi-confined concrete, and post-confined concrete. The extension of GFRP is more significant than that of CFRP, which is related to the elastic modulus of GFRP. The enhancement ratio of FRP semi-confined concrete is about half of that of fully confined concrete. Post-confined concrete has a slight increase in extension compared to un-eroded confined concrete.

**Table 5:** The ultimate stress and strain.

Type	F/kN	f ′_cc_/MPa	f′_cc_/f′_co_	ε′_cc_	ε′_h_	ε′_cc_/ε′_co_
U-FT00	180.6	23	1	1840	673	1
U-FT50	225.3	28.7	1	2390	797	1
U-FT100	190.0	24.2	1	3110	1040	1
CF-FT00	614.7	78.3	3.4	16,317	12,985	8.9
CF-FT50	632.7	80.6	3.5	17,755	14,055	9.6
CF-FT100	663.3	84.5	3.6	17,929	14,517	9.7
GF-FT00	366.6	46.7	2.0	17,665	15,500	9.6
GF-FT50	393.3	50.1	2.2	18,549	16,275	10
GF-FT100	457.7	58.3	2.5	19,432	17,050	10.6
CS-FT00	412.1	52.5	2.3	7572	14,550	4.1
CS-FT50	439.6	56	2.4	7970	15,316	4.3
CS-FT100	423.9	54	2.3	8209	17,903	4.5
GS-FT00	277.1	35.3	1.5	9138	18,589	5.0
GS-FT50	328.1	41.8	1.8	10,664	20,607	5.8
GS-FT100	281.8	35.9	1.6	11,197	1638	6.1
FT50-CF	505.5	64.4	2.2	13,241	11,285	5.5
FT50-GF	404.3	51.5	1.8	19,629	18,257	8.2
FT50-CS	351.7	44.8	1.6	7730	14,728	3.2
FT50-GS	317.1	40.4	1.4	10,231	21,632	4.3
FT50-CF-FT50	521.2	66.4	2.7	14,872	12,291	4.8
FT50-GF-FT50	398.0	50.7	2.1	20,610	19,170	6.6
FT50-CS-FT50	396.4	50.5	2.1	8137	15,498	2.6
FT50-GS-FT50	325.8	41.5	1.7	10,646	21,634	3.4

ε′_cc_ is the axial strain at the peak point of the ordinary FRP confined concrete; ε′_h_ is the lateral strain at the peak point of the ordinary FRP confined concrete; ε′_co_ is the axial strain at the peak point of the unconfined concrete. f′_cc_ is the axial stress at the peak point of the ordinary FRP confined concrete; f′_co_ is the axial stress at the peak point of the unconfined concrete.

Numerous researchers have proposed an ultimate stress–strain model for FRP-confined concrete subjected to erosion. However, the model has not been studied in a freeze–thaw cycle environment with various confined materials and technologies in a sodium sulfate solution. The ultimate stress–strain calculation models for FRP-confined concrete developed by the four researchers are shown in Table 6, with standardized parameter symbols for each model. The ultimate stress–strain method determined the best fit model using error analysis. The four models for predicting ultimate stress and strain are based on strongly confined concrete. By observing the stress–strain curve of concrete in Fig. 13, determine its ultimate stress and strain. The stress of FRP-confined concrete increases with increasing strain, and there is no descending segment. The results show that the peak strain and ultimate strain of FRP-confined concrete are consistent. The ultimate strain of unconfined concrete is the strain corresponding to the ultimate stress.

**Table 6:** FRP ultimate stress–strain model.

Model type	Karbhari & Gao model^32^	Toutanji model^33^	Lam & Teng model^34^	Jiang & Teng model^35^
Formula 1	$\frac{{{\text{f}}_{{{\text{cc}}}} }}{{{\text{f}}_{{{\text{co}}}} }}{ = 1 + 2}{\text{.1(}}\frac{{{\text{f}}_{{\text{l}}} }}{{{\text{f}}_{{{\text{co}}}} }}{)}^{{{0}{\text{.87}}}} \,$	$\frac{{{\text{f}}_{{{\text{cc}}}} }}{{{\text{f}}_{{{\text{co}}}} }}{ = 1 + 3}{\text{.5}} \times {(}\frac{{{\text{f}}_{{\text{l}}} }}{{{\text{f}}_{{{\text{co}}}} }}{)}^{{{0}{\text{.85}}}} \,$	$\frac{{{\text{f}}_{{{\text{cc}}}} }}{{{\text{f}}_{{{\text{co}}}} }}{ = 1 + 2}{\text{.0(}}\frac{{{\text{f}}_{{\text{l}}} }}{{{\text{f}}_{{{\text{co}}}} }}{)}^{{}} \,$	$\frac{{{\text{f}}_{{{\text{cc}}}} }}{{{\text{f}}_{{{\text{co}}}} }}{ = 1 + 3}{\text{.5(}}\frac{{{\text{f}}_{{\text{l}}} }}{{{\text{f}}_{{{\text{co}}}} }}{)}^{{}} \,$
Formula 2	${\upvarepsilon }_{{{\text{cc}}}} {{ = \varepsilon }}_{{{\text{co}}}} { + 0}{\text{.01}}\left( {\frac{{{\text{f}}_{{\text{l}}} }}{{{\text{f}}_{{{\text{co}}}} }}} \right)^{{}} \,$	$$\begin{gathered} \frac{{{\upvarepsilon }_{{{\text{cc}}}} }}{{{\upvarepsilon }_{{{\text{co}}}} }}{ = 1 + }\left( {{310}{{.57\varepsilon }}_{{\text{l}}} + 1.90} \right) \hfill \\ \left( {\frac{{{\text{f}}_{{{\text{cc}}}} }}{{{\text{f}}_{{{\text{co}}}} }} - 1} \right) \hfill \\ \end{gathered}$$	$\frac{{{\upvarepsilon }_{{{\text{cc}}}} }}{{{\upvarepsilon }_{{{\text{co}}}} }}{ = 2 + }1{5}\left( {\frac{{{\text{f}}_{{\text{l}}} }}{{{\text{f}}_{{{\text{co}}}} }}} \right)$	$\frac{{{\upvarepsilon }_{{{\text{cc}}}} }}{{{\upvarepsilon }_{{{\text{co}}}} }}{ = 1 + }1{7}{\text{.5}}\left( {\frac{{{\text{f}}_{{\text{l}}} }}{{{\text{f}}_{{{\text{co}}}} }}} \right)^{1.2}$

f_cc_ is the compressive strength of the FRP-confined concrete specimen; f_co_ is the compressive strength of the unconfined concrete specimen; f_l_ is the lateral confined strength provided by FRP; ε_cc_ is the limit point strain corresponding to the FRP-confined concrete; ε_co_ is the unconfined concrete The limit point strain corresponding to the unconfined concrete.

**Figure 14:**
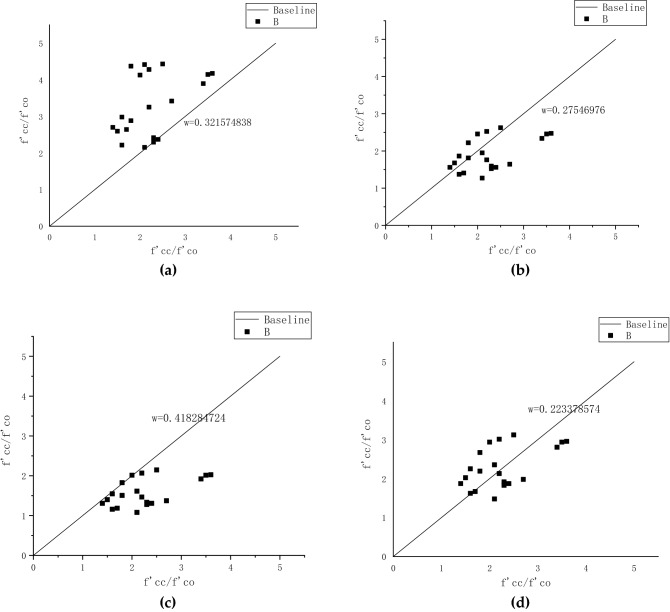
Error analysis of ultimate stress of FRP-confined concrete: **(a)** Karbhari & Gao model error analysis; **(b)** Toutanji model error analysis; **(c)** Lam & Teng model error analysis; **(d)** Jiang & Teng model error analysis.

The actual ε′_cc_/ε′_co_ values in Table 4 are substituted into the above research model, and the predicted values of f′_cc_/f′_co_ are calculated. It is compared with the actual f′_cc_/f′_co_ value for the enhancement ratio error analysis in Fig. 14a. The abscissa represents the actual enhancement ratio, and the ordinate represents the predicted enhancement ratio. The results show that the predicted enhancement ratio of the Karbhari & Gao model is larger than the actual enhancement ratio. Figure 14b shows that the baseline of the Toutanj model is at the center of the scatter, with relatively small errors. Figure 14c illustrates that the predicted enhancement ratio is smaller than the actual enhancement ratio. The error bounds of the Jiang & Teng model are comparable to those of the Toutanji model in Fig. 14d. It is concluded that the Jiang & Teng and Toutanji model is most similar to the model under this experimental condition. Finally, the error rate w($\frac{{\sum {{\text{(A}} - {\text{A}^{\prime}}} )}}{{\sum {\text{A}} }}$, where A' is the predictive value and A is the test value)is introduced.

**Figure 15:**
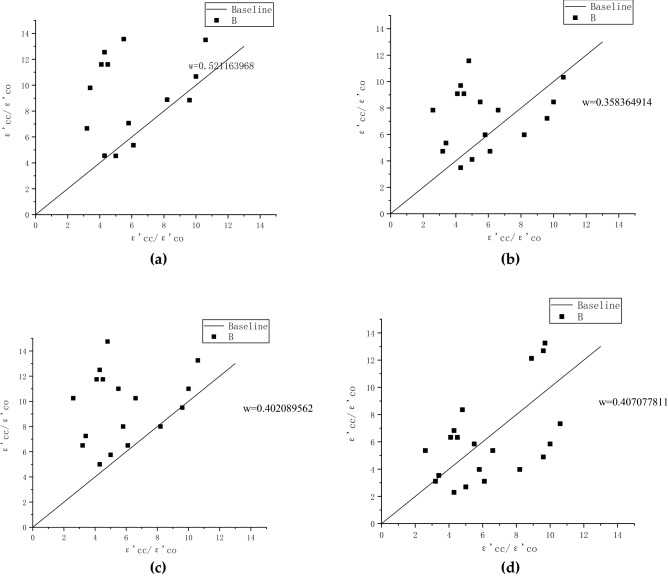
Error analysis of ultimate strain of FRP-confined concrete: **(a)** Karbhari & Gao model error analysis; **(b)** Toutanji model error analysis; **(c)** Lam & Teng model error analysis; **(d)** Jiang & Teng model error analysis.

The actual f′_cc_/f′_co_ values in Table 4 are substituted into the above research model, and the predicted values of ε′_cc_/ε′_co_ are calculated. In Fig. 15, the abscissa represents the actual extension ratio, and the ordinate represents the calculated predicted extension ratio. Figure 15a illustrates that the predicted extension ratio of the Karbhari & Gao model is larger than the actual. The baseline of the Toutanji model is located at the center of the scatter, and the error is smaller in Fig. 15b. Figure 15c shows that the calculated extension ratio is larger than the actual extension ratio. The error range of the Jiang & Teng model shown in Fig. 15d is comparable to that of the Toutanji model. Combined with the enhancement ratio error analysis, it is concluded that the Jiang & Teng model is the closest to the experimental conditions.

**Figure 16:**
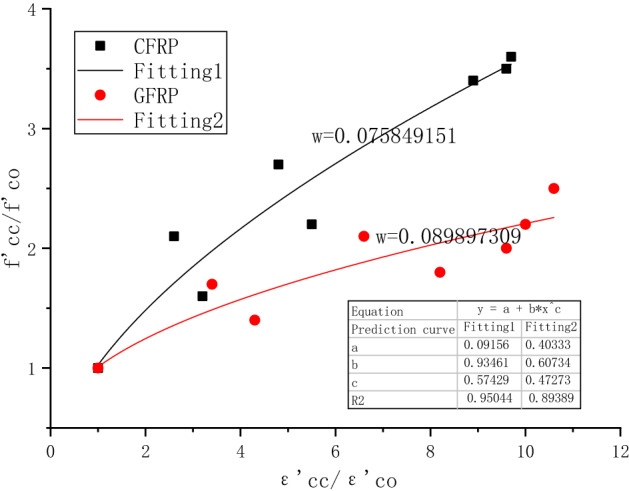
CFRP and GFRP-confined concrete prediction models.

The previous limit stress–strain prediction model shows that the current research model can not meet the prediction when FRP-confined concrete is subjected to freeze–thaw cycles in a sodium sulfate solution. Therefore, a prediction model under this condition is established in this experiment based on the obtained data. This presented model has limited applicability and can only predict within 10% mass sodium sulfate solution and 100 freeze–thaw cycles. Exceeding this range requires more experiments for refinement. The test results are divided into two categories, namely CFRP-confined concrete and GFRP-confined concrete. The error rates of the prediction models are 0.075 and 0.089, respectively. All analysis results show that the model has a good prediction effect.

6 $$\frac{{{{{\rm f}^{\prime}}}_{{{\text{cc}}}} }}{{{{{\rm f}^{\prime}}}_{{{\text{co}}}} }}= {\text{ a }} + {\rm b} \left( {\frac{{{{\varepsilon ^{\prime}}}_{{{\text{cc}}}} }}{{{{\varepsilon ^{\prime}}}_{{{\text{co}}}} }}} \right)^{{\text{c}}}$$

where f_cc_ is the compressive strength of the FRP-confined concrete specimen; f_co_ is the compressive strength of the unconfined concrete specimen; ε_cc_ is the limit point strain corresponding to the FRP-confined concrete; ε_co_ is the unconfined concrete. The limit point strain corresponding to the unconfined concrete. a, b, and c are the values to be fitted, and their values are shown in Fig. 16.

## Conclusion

Experiments are conducted to determine the effect of freeze–thaw cycles on FRP-confined concrete in a sulfate solution. External environmental conditions use sodium sulfate at 10% concentration and freeze–thaw cycles. The following conclusions are drawn following microscope observation, pH, mass loss, SEM and EDS analysis, axial compressive strength testing, and stress–strain curve analysis.

1. The more freeze–thaw cycles, the more serious the surface damage, which gradually erodes from the surface to the inside. FRP semi-confined and unconfined surfaces are severely damaged, while FRP fully-confined surfaces are barely detectable. Semi-confined technology does not reduce the chemical attack of the environment on the concrete, but it can ensure the corresponding strength.

2. The pH of unconfined concrete increases rapidly during erosion and gradually increases over time. EDS spectroscopy is used on the specimen to analyze further the white crystals observed in the SEM. Sodium sulfate reacts with calcium hydroxide to form calcium sulfate, and calcium sulfate reacts with calcium meta aluminate to form alum crystals. The predicted relationship between pH and mass loss under different confined techniques is established.

3. The mechanical test confirms that FRP confined technology significantly improves the ultimate load of concrete. FRP semi-closed technology can achieve about 50% of the effect of fully closed technology. The mechanical effect of CFRP confinement is twice that of GFRP confinement. Under the action of coupled erosion, the strength of FRP semi-compressed and uncompressed concrete first increased and then decreased slightly. The strength of FRP fully restrained concrete shows a slow increase. Furthermore, concrete that has been eroded and then confined retains a high level of performance when eroded again. A predictive model of the relationship between mass loss and strength loss is built.

4. The stress–strain curves of semi-confined and fully-confined concrete can be divided into three stages. The ultimate stress–strain model for FRP-confined concrete is validated by referring to previously published literature. A new model is presented to predict the ultimate strength under the coupled erosion of freeze–thaw and sulfate. It can predict the ultimate stress–strain of concrete confined by two materials, CFRP and GFRP, under the coupled conditions of freeze–thaw in a sulfate solution.

## Data Availability

All data generated or analysed during this study are included in this published article.
